# The Mechanical Stimulation of Myotubes Counteracts the Effects of Tumor-Derived Factors Through the Modulation of the Activin/Follistatin Ratio

**DOI:** 10.3389/fphys.2019.00401

**Published:** 2019-04-24

**Authors:** Alexandra Baccam, Alexandra Benoni-Sviercovich, Marco Rocchi, Viviana Moresi, Marilia Seelaender, Zhenlin Li, Sergio Adamo, Zhigang Xue, Dario Coletti

**Affiliations:** ^1^Biology of Adaptation and Aging (B2A), Sorbonne Université, UMR8256 – INSERM ERL U1164, Paris, France; ^2^Section of Histology, Department of Anatomical, Histological, Forensic and Orthopedic Sciences, Sapienza University of Rome, Rome, Italy; ^3^Interuniversity Institute of Myology, Rome, Italy; ^4^Department of Biomolecular Sciences, University of Urbino, Urbino, Italy; ^5^Institute of Biomedical Sciences, Faculdade de Medicina, University of São Paulo, São Paulo, Brazil

**Keywords:** skeletal muscle atrophy, myokines, mechanotransduction, exercise, C26 colon carcinoma, FlexCell apparatus

## Abstract

Activin negatively affects muscle fibers and progenitor cells in aging (sarcopenia) and in chronic diseases characterized by severe muscle wasting (cachexia). High circulating activin levels predict poor survival in cancer patients. However, the relative impact of activin in mediating muscle atrophy and hampered homeostasis is still unknown. To directly assess the involvement of activin, and its physiological inhibitor follistatin, in cancer-induced muscle atrophy, we cultured C2C12 myotubes in the absence or in the presence of a mechanical stretching stimulus and in the absence or presence of C26 tumor-derived factors (CM), so as to mimic the mechanical stimulation of exercise and cancer cachexia, respectively. We found that CM induces activin release by myotubes, further exacerbating the negative effects of tumor-derived factors. In addition, mechanical stimulation is sufficient to counteract the adverse tumor-induced effects on muscle cells, in association with an increased follistatin/activin ratio in the cell culture medium, indicating that myotubes actively release follistatin upon stretching. Recombinant follistatin counteracts tumor effects on myotubes exclusively by rescuing fusion index, suggesting that it is only partially responsible for the stretch-mediated rescue. Therefore, besides activin, other tumor-derived factors may play a significant role in mediating muscle atrophy. In addition to increasing follistatin secretion mechanical stimulation induces additional beneficial responses in myotubes. We propose that in animal models of cancer cachexia and in cancer patients purely mechanical stimuli play an important role in mediating the rescue of the muscle homeostasis reported upon exercise.

## Introduction

Cancer cachexia is multifactorial and characterized by tumor- and host-derived factors leading to muscle wasting (Fearon et al., 2012). TGF-β family members, including activin and myostatin, are key regulators of muscle development and homeostasis (Chen et al., 2016) and have been reported to mediate cachexia (Costelli et al., 2008; Chen et al., 2014). Since they bind to activin type IIB receptor (ActRIIB), the latter has been targeted to counteract muscle wasting (Barreto et al., 2016; Hatakeyama et al., 2016; Toledo et al., 2016) or to promote muscle hypertrophy (Morvan et al., 2017) and the regenerative capacity of muscle (Formicola et al., 2018). Activin induces muscle catabolism via p38β (Ding et al., 2017) and SMAD2/3 activation (Winbanks et al., 2016). Chemotherapy activates this pathway too, further worsening muscle atrophy (Barreto et al., 2016; Coletti, 2018). Follistatin (or activin-binding protein) is a potent, physiological inhibitor of activin and myostatin (Zheng et al., 2017). Several organs such as the gonads (Tilbrook et al., 1996) and skeletal muscle (Ciaraldi et al., 2016) are sources of follistatin.

Exercise modulates follistatin and other myokines (Yeo et al., 2012) and the plasma profile of cytokines (Lira et al., 2009; Donatto et al., 2013), producing marked beneficial effects on muscle homeostasis (Barone et al., 2016; Coletti et al., 2016; Pigna et al., 2016). This is the reason why exercise is recommended to treat cachexia (Lira et al., 2012, 2014).

Even though a high level of circulating activin is an adverse prognostic factor in cancer patients (Loumaye et al., 2017), it is yet unknown if activin is a direct player or only a mere marker of cachexia. Besides, it is not known if beneficial exercise effects are mediated by purely mechanical stimuli in the muscle, nor whether exercise itself modulates the follistatin/activin ratio and its paracrine consequences. To address these questions, we cultured C2C12 myotubes in the absence or in the presence of mechanical stretching, and in the absence or in the presence of tumor-derived factors, so as to mimic the mechanical stimulation of exercise and cancer cachexia, respectively.

## Materials and Methods

### Cell Cultures

C2C12 were seeded at a density of 20000 cells/cm^2^ in the multiwell plate of the Flexcell^®^ FX-6000^TM^ Tension System designed for unidirectional stretching (collagen coated silicon membrane, 3.89 cm^2^). A vacuum was constantly applied to stretch the membrane by 6%, in order to increase membrane stiffness, a condition that preliminary experiments showed to be necessary to allow a proper differentiation of the C2C12 cells into myotubes (data not shown). The following day, at 80% confluence, GM (DMEM with 15% FBS, 4.5 g/L of glucose, 2 mM of L-glutamine, 100 μg/mL of penicillin-streptomycin; Sigma-Aldrich, St. Louis, MO, United States) was replaced with DM containing 2% Horse Serum (HS, Carotenuto et al., 2016). A mixed culture of myoblasts and myotubes was kept for 4 days in DM at 37°C, 5% of CO2 in the Flexcell apparatus, always under constant stretch. For the experiments, the initial myotubes cultures were further cultured for additional 2d under continuous stretching (SC, Static Condition) or were subjected to 2 daily series of 2 h cyclic stretching (0,5 Hz, 6% stretch), with a 3 h-pause between them (DC, Dynamic Condition). As to media composition during the 2d experimental treatments see figure legends (Sigma reagents).

In order to obtain a tumor cell conditioned medium (CM), C26 carcinoma cells (Cell Lines Service) were cultured for 2d in serum-free DMEM and the supernatant being diluted to 20% in HS medium. To obtain the control medium, DMEM was incubated for 2d at 37°C in the absence of C26 cells.

### Immunofluorescence, ELISA

Samples underwent standard procedures (De Arcangelis et al., 2005; Aulino et al., 2015). Antibodies: MF20 anti-MHC and F5D anti-myogenin (DSHB); Alexafluor 555 or 488 secondary Abs (Molecular probes). Regarding the ELISA quantification of some specific factors secreted in C26 CM or in the myotube culture media DAC00B Human/Mouse Activin A and DFN00 Human Follistatin (R&D) systems were used following manufacturer’s instructions.

### Western Blot

Protein extraction and electrophoresis were performed as previously described (Coletti et al., 2016). In brief: samples were treated with lysis buffer RIPA containing Tris-Cl 50 Mm pH = 7.5, 150 Mm NaCl, 1% NP40, 0.5% desoxyclorate de sodium, EGTA 20 mM, DTT 1 mM, and a protease inhibitor cocktail. Proteins were denatured with a Bolt kit (Molecular probes, Invitrogen). Total protein content was measured by Bradford and GAPDH was used as a loading control. Membranes of nitrocellulose were incubated with blocking buffer TBS-Tween with 5% not fat milk. Antibodies: anti-MyoD, anti Phospho-SMAD2/3, anti-SMAD2/3 (Cell Signalling), anti-GAPDH (Sigma). The sample size was 9, however, triplicate independent samples were pooled to obtain a sufficient amount of proteins to be loaded in each lane. Secondary antibody fluorescence was detected by using the Odyssey system; for each band, quantification of the signal was obtained by ImageJ, following background subtraction, and each value was normalized by the corresponding GAPDH band intensity; for SMADs, the ratio between the normalized P-SMAD and SMAD values was calculated.

### Quantitative PCR

Total RNA was isolated with Trizol^®^ reagent (Invitrogen) following the manufacturer’s recommendations and homogenized. RNA concentration was determined by measuring the absorbance in 260 nm/280 nm in a NanoDrop spectrophotometer. cDNA synthesis was carried out using the High capacity applied Reverse Transcription Kit (Biosystem). Lightcycler 480 was used to detect SYBR Green signal in Q-PCR. The mRNA levels were determined by the comparative Ct method; the average ΔCt value of the control group was subtracted from the test value to derive a −ΔΔCt value. The expression of each gene was evaluated by 2^−ΔΔCt^, according to Livak and Schmittgen (2001). List of primers used:

ActRIIBL: CTG-TGC-GGA-CTC-CTT-TAA-GC
ActRIIBR: TCT-TCA-CAG-CCA-CAA-AGT-CG
Activin-AL: CAG-TGG-GGA-GGT-CCT-AGA-CA
Activin-AR: CAA-AAG-GAG-CAG-CAG-AGA-CC
FollistatinL: CCT-CCT-GCT-GCT-GCT-ACT-CT
FollistatinR: TGC-TGC-AAC-ACT-CTT-CCT-TG
Wnt4L: CTG-GAG-AAG-TGT-GGC-TGT-GA
Wnt4R: GGA-CGT-CCA-CAA-AGG-ACT-GT
MyoDL: GAG-ATG-CGC-TCC-ACT-ATG-CT
MyoDR: TGG-CAT-GAT-GGA-TTA-CAG-CG
MyogeninL: GCA-CTG-GAG-TTC-GGT-CCC-AA
MyogeninR: TAT-CCT-CCA-CCG-TGA-TGC-TG

### Imaging and Morphometry

Images were acquired by a Zeiss EM S3/SyCoP3 Macro-apotome equipped with Zen software in the imaging facility of the Institute of Biology Paris-Seine. The ImageJ software was used for the morphometric analysis. Fusion index (FI) was defined as the number of nuclei in myotubes on total nuclei in 5 fields/sample; myotube diameter (DIA) was measured in 50 myotubes/sample; nuclei per myotube (NpM) were counted in 50 randomly chosen myotubes. Myotubes diameter was measured as the average from three independent measurements per myotube according to previously published methods (Trendelenburg et al., 2009; Deane et al., 2013). At least triplicate samples from two independent experiments were analyzed for each condition; thus, 6 < *n* < 10 for each data group.

### Statistical Analysis

Comparisons of quantitative variables were performed through 2-way ANOVA, after verifying parametric assumptions. In case these assumptions were violated, some transformations (square root or arcsin, as appropriate) were used. *Post hoc* comparisons were performed through Tukey’s significant difference method. When a comparison of each treatment group with a single control group was necessary, a Dunnett *post hoc* test was employed. The significance level was set at 0.05. Statistical analyses were performed by SPSS 25.0.

## Results

### Mechanical Stimulation Counteracts the Negative Effect of Tumor-Derived Factors on Muscle Cells

**FIGURE 1:**
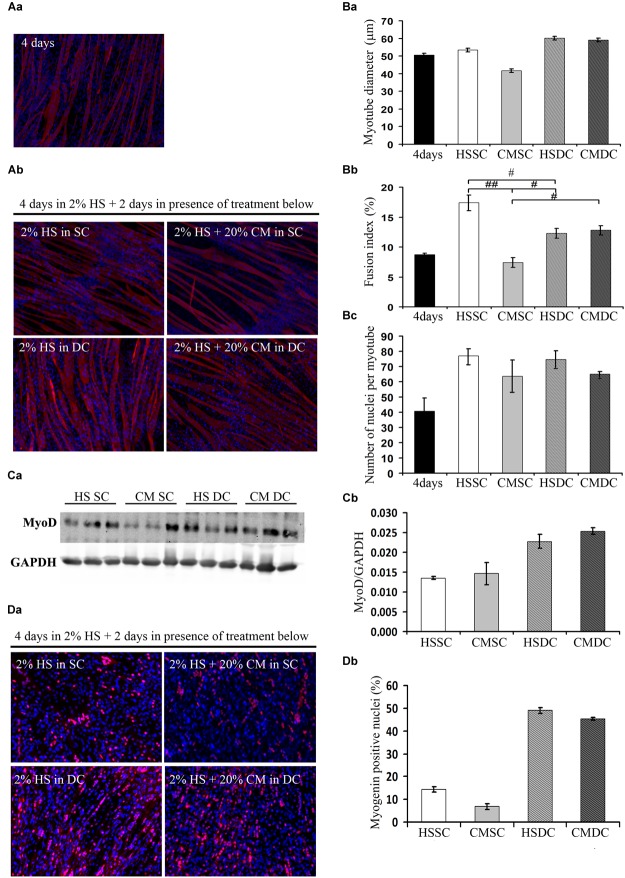
Mechanical stimulation counteracts the negative effect of tumor-derived factors. **(A)** Myosin (red) localization and nuclei (blue) by immunofluorescence in C2C12 myotubes at 4d **(Aa)** and 6d **(Ab)** of culture in a differentiation medium in the absence (HS) or presence (CM) of C26-conditioned medium, in combination with the absence (SC) or presence (DC) of cyclic stretching. **(B)** Morphometric analyses were performed on replicate samples (*n* = 6). One-way ANOVA performed on data from 4d and 6d (five groups) followed by Dunnet’s test indicated a significant increase in the fusion index (FI) and in the number of nuclei/myotube (NpM) between 4d and 6d in any condition except CM. Two-way ANOVA performed on 6d values showed a significant effect for: **(Ba)** DC on myotube diameter (*F* = 12.66; *df* = 1; *p* < 0.05); **(Bb)** CM and interaction with DC (for CM: *F* = 24.73; *df* = 1; *p* < 0.001; for interaction: *F* = 30.2; *df* = 1; *p* < 0.001) on fusion index; ^#^*p* < 0.05, ^##^*p* < 0.01 by Tukey HSD test); **(Bc)** CM on number of nuclei/myotube (*F* = 5.64; *df* = 1; *p* < 0.05). **(C)** WB analysis for MyoD **(Ca)** and relative average density **(Cb)** following normalization over the GAPDH signal in the same conditions as above. Two-way ANOVA showed a significant effect for DC (*F* = 19.47; *df* = 1; *p* < 0.001). **(D)** Myogenin (red) and nuclei (blue) by IF **(Da)** in C2C12 myotubes at 6d of culture in the same conditions as above, and quantification of the percentage of myogenin+ nuclei **(Db)**. Two-way ANOVA showed the significant effect of both CM (*F* = 13.55; *df* = 1; *p* < 0.01) and DC (*F* = 451.7; *df* = 1; *p* < 0.0001), indicating that the DC rescues myogenin expression. Data are shown as mean ± SEM.

C2C12 cultures, following 4d in DM, contained both multinucleated myotubes and undifferentiated myoblasts (Figure 1Aa). We further cultured these cells for 2d in control conditions (i.e., in HS) in the absence (static condition, SC) or presence (dynamic condition, DC) of mechanical stimulation, represented by cyclical stretching of the substratum; furthermore, we treated the cells with C26 tumor-conditioned medium (CM), in a SC or a DC, and we analyzed 6d cultures undergoing four combinatorial treatments (Figure 1Ab). The morphometric analysis focused on myotube diameter (DIA), as a marker of fiber size, on fusion index (FI), as a marker of the extent of myogenic differentiation, and on the number of nuclei per myotube (NpM), as an indication of myotube growth because of the addition of nuclei deriving from the myoblasts. On day 6 myotube cultures showed a significant increase in FI and NpM as compared to 4d cultures, indicating that the myotubes continuously grew in size by incorporating the nuclei from myoblasts, or, possibly, that additional newborn myotubes formed (Figure 1B). Two-way ANOVA on 6d-culture morphological features showed that: CM decreased, while DC significantly increased, myotube DIA even in the presence of CM (Figure 1Ba); CM diminished FI, while DC interfered with CM and rescued FI. Given the significance of the negative interaction between CM and DC we could perform *post hoc* tests, which showed not only that the FI in the presence of CM is lower compared to all the other treatments, but also that the DC does not promote fusion *per se* (Figure 1Bb); indeed CM had a negative effect on the number of NpM, with no interaction with the DC, while the latter did not significantly affect the number of NpM (Figure 1Bc).

Consistently with the previous results, the positive effects of mechanical stimulation on myogenesis were confirmed by the significant upregulation of the MyoD level (Figure 1Ca), likely mirroring a sustained activation of differentiating myoblasts; we also observed a decreased, albeit not significant, amount of MyoD protein following CM treatment, with no interaction between the two variables as shown by ANOVA (Figure 1Cb). The immunofluorescence analysis of myogenin (Figure 1Da), which is a later differentiation marker and is required for terminal myogenic differentiation, revealed statistically significant, opposite effects of both CM and DC (Figure 1Db) on the percentage of the myogenin positive nuclei.

### Follistatin Is Sufficient to Rescue Myogenic Differentiation but Not Myotube Size in the Presence of Tumor-Derived Factors

**FIGURE 2:**
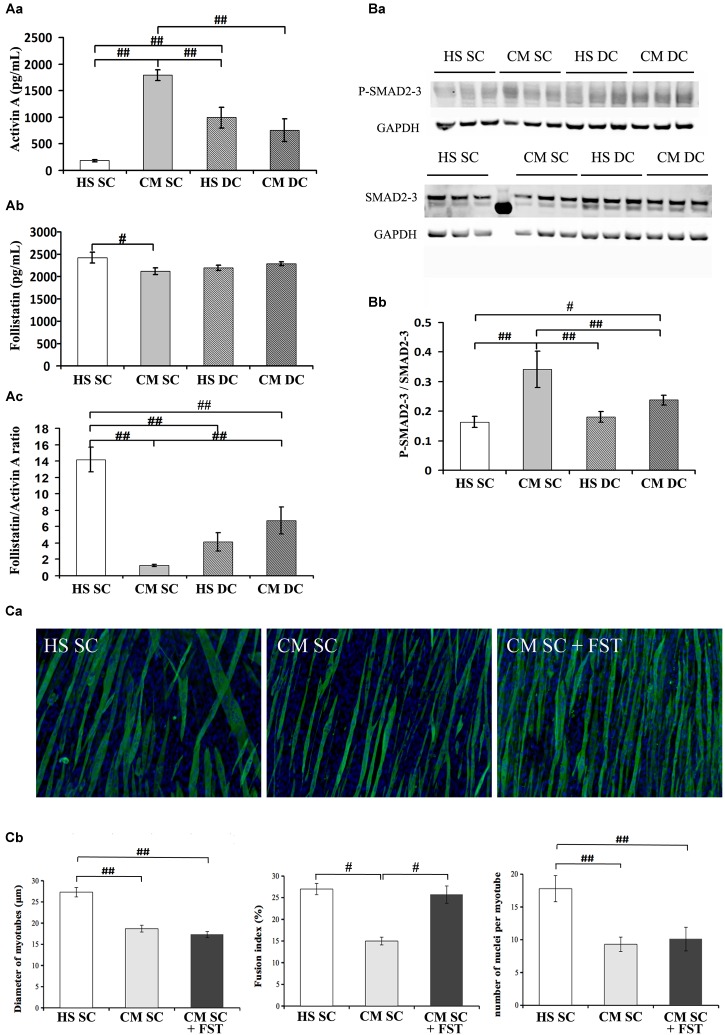
Follistatin is not sufficient to rescue myotube size in the presence of tumor-derived factors. **(A)** Quantification of activin **(Aa)** and follistatin **(Ab)** by ELISA in 6d culture supernatant, in differentiation medium in the absence (HS) or presence (CM) of C26-conditioned medium, in combination with the absence (SC) or presence (DC) of cyclic stretch. The follistatin/activin ratio was calculated **(Ac)**. Two-way ANOVA showed: CM effect and a interaction with DC on activin levels; interaction between CM and DC on follistatin levels; CM effect and interaction with DC on follistatin/activin ratio. ^#^*p* < 0.05, ^##^*p* < 0.01 by Tukey HSD test. **(B)** WB analysis for P-SMAD2/3 and SMAD 2/3 **(Ba)** and relative average density **(Bb)** following normalization over the GAPDH. Two-way ANOVA showed a significant effect for DC (*F* = 6.25; *df* = 1; *p* < 0.05) and interaction of Dc with CM (*F* = 12.36; *df* = 1; *p* < 0.01). ^#^*p* < 0.05, ^##^*p* < 0.01 by Tukey HSD test. **(C)** Immunofluorescence for Myosin (green) and nuclei (blue) in C2C12 myotubes at 6d of culture **(Ca)** in differentiation medium in the absence (HS) or presence (CM) of C26-conditioned medium and in CM supplemented with 100 ng/ml recombinant follistatin, in the absence of cyclic stretch (SC). The treatments were performed starting on 4d culture and changing the medium daily. Morphometric analyses **(Cb)** were performed on replicate samples (*n* = 6). One-way ANOVA indicated a significant effect of treatments on myotube diameter and on the number of nuclei/myotube. ^∗∗^*p* < 0.01 by Tukey HSD test.

To assess the contribution of myokines and putative tumor-derived factors on myotube size and myoblast recruitment, we measured both activin and follistatin levels in the 6d culture media. Worth noting, the C26 CM used throughout this work contained 2800 ± 380 pg/mL activin (data not shown), implying that, following a dilution to 20% in the culture medium, the latter contained 590 ± 76 pg/mL activin of tumor origin at the beginning of the treatments, i.e., on day 4 (data not shown). Following 2 days in culture, activin concentration decreased to 190 ± 18 pg/mL in control conditions (HS SC) indicating a non-specific or myotube-mediated degradation or internalization/absorption (Figure 2Aa); on the contrary, in the presence of CM activin increased about 3 times, which suggests an activin release from muscle cells (Figure 2Aa). In the DC activin levels were significantly reduced, both in the absence or presence of CM, even though they remained higher than in unstimulated, control cultures (Figure 2Aa).

In order to better understand the novel finding of the release of activin from muscle cells, we measured activin A expression in the four culture conditions failing to see any statistically significant difference, even though we noticed that activin expression doubled in CM SC (Table 1); this suggests that activin release from the myotubes could be partially dependent on activin expression but mostly depends on post-translational events.

**Table 1:** Mechanical stimulation counteracts the negative effect of tumor-derived factors on P-SMAD transcriptional targets (MRF) and on Follistatin expression.

Gene	HS SC	CM SC	HS DC	CM DC	ANOVA
ActRIIB	1.00 ± 0.36	5.35 ± 1.52	1.56 ± 0.51	0.99 ± 0.14	DC, CM QS interaction
Activin A	1.00 ± 0.30	2.02 ± 0.53	1.21 ± 0.38	1.18 ± 0.29	NS
Follistatin	1.00 ± 0.17	0.50 ± 0.09	1.77 ± 0.43	1.02 ± 0.25	DC and CM effects
Wnt4	1.00 ± 0.27	2.70 ± 0.74	1.58 ± 0.36	1.23 ± 0.27	interaction
MyoD	1.00 ± 0.09	0.44 ± 0.05	0.70 ± 0.15	0.66 ± 0.15	DC and CM effects interaction
Myogenin	1.00 ± 0.16	0.59 ± 0.08	0.97 ± 0.08	0.92 ± 0.11	DC and CM effects

*Gene expression was assessed by Q-PCR in myotubes following 2d culture as indicated in the absence (HS) or presence (CM) of C26-conditioned medium, in combination with the absence (SC) or presence (DC) of cyclic stretch. Gene expression is shown as fold induction in respect to control (HS SC), following normalization over GAPDH. Data are shown as mean ± SEM of replicate samples (8 < n < 16). Two-way ANOVA significance is reported in the last column (ANOVA) and ANOVA results are reported in parentheses for each gene, as follows: ActRIIB, activin Receptor IIB (for DC and for CM: Q.S.; interaction F = 8.67; df = 1; p < 0.01); activin A (NS); Follistatin (for DC: F = 6.71; df = 1; p < 0.05; for CM: F = 6.09; df = 1; p < 0.05); Wingless-related integration site 4, Wnt4 (F = 4.74; df = 1; p < 0.05); MyoD (For DC: F = 12.58; df = 1; p < 0.01; for CM: F = 6.85; df = 1; p < 0.05; interaction: F = 6.43; df = 1; p < 0.05); myogenin (for DC: F = 3.97; df = 1; p < 0.05; for CM: F = 4.42; df = 1; p < 0.05; interaction: QS). NS and QS = not and quasi (p = 0.05) significant, respectively.*

We also measured follistatin concentration in the four conditions above and noticed that it significantly decreased in the CM SC (Figure 2Ab), consistently with the physiological role of follistatin as an activin-binding protein, and that its level was rescued in the presence of mechanical stimulation, i.e., DC. Worth noting, the CM and the DC have opposite, significant effects on follistatin expression (Table 1), indicating that the exposure to tumor-derived factors, including activin, downregulates follistatin production in muscle cells, while the mechanical stimulation rescues follistatin expression and release.

Since free activin binds to the activin receptor type-2B (actRIIB) we could not exclude that mechanical stimulation counteracted CM effects by affecting the actRIIB expression as well. Therefore, we measured actRIIB receptor expression in muscle cell cultures and we found a quasi-significant effect of both CM and DC on its expression. In addition, we found that mechanical stimulation interferes with the CM-induced actRIIB expression increase, further contributing to the myotube desensitization to activin (Table 1). Altogether, these data suggest an adverse effect on myotubes of tumor cell- and muscle-derived activin, which could be counteracted by the mechanically stimulated secretion of follistatin by myotubes (Figure 2A). Activin effects are further exacerbated by the differential modulation of actRIIB by CM and DC (Table 1).

Given the pivotal role of actRIIB in mediating cachexia *in vivo* (Zhou et al., 2010) and the negative effects of tumor-derived factors on myotubes reported above, we aimed to confirm that the ActRIIB signaling pathway was differentially activated by CM and DC. Therefore, we measured SMAD2/3 activation (expressed as P-SMAD2/3 over total SMADs, following normalization by the housekeeping gene GAPDH, Figure 2Ba) and found consistent results, i.e., SMAD2/3 activation by the CM and inhibition by the DC (Figure 2Bb). To further confirm these results, we analyzed SMAD2/3 transcriptional targets, including Wnt4 and, noticeably, the MRF MyoD and myogenin (Table 1). As expected, a negative interaction was found between DC and CM on Wnt4 expression: the latter was increased by the CM, albeit not significantly (possibly a false negative result in this case); in addition, a return to control levels was observed in the DC (Table 1). As is known, SMAD2/3 transcriptional effects on MRF are the opposite than those on Wnt4, since their expression is inhibited by P-SMAD2/3: so, as expected and in line with the protein levels shown in Figure 1, the CM significantly decreased both MyoD and myogenin expression, while the DC restored MyoD and myogenin expression to control levels (Table 1).

The correlation between a high follistatin/activin ratio and the improvement of myogenesis prompted us to investigate whether follistatin was sufficient to counteract CM effects upon myotubes. To this purpose, we incubated myotube cultures in SC with CM in the absence or presence of recombinant follistatin (Figure 2Ca). While CM decreased myotube DIA and hampered FI and NpM increase, follistatin rescued FI but failed to counteract CM effects on DIA and NpM (Figure 2Cb). Worth noting, in a control experiment recombinant follistatin alone was able to counteract the adverse effects of recombinant activin upon myotube DIA and FI, as a proof of concept of its inhibitory activity on activin (Supplementary Figure S1).

## Discussion

**FIGURE 3:**
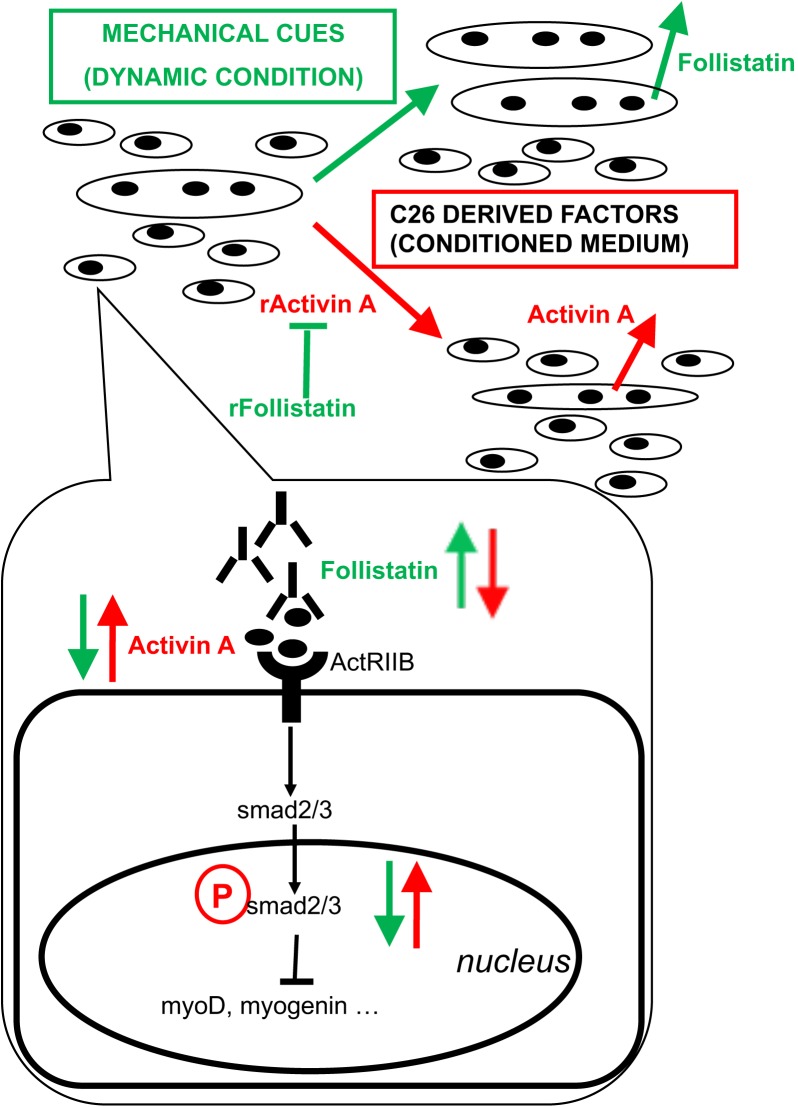
Proposed model of action of tumor-derived factors and mechanical stimulation on myotubes and myoblasts. Mixed cultures of myotubes and myoblasts mature in culture by increasing the diameter of myotubes, the fusion index (i.e., myogenic differentiation *tout court*, including the formation of novel myotubes) and the number of nuclei per myotube (i.e., myotube accretion by incorporation of myoblasts). C26 tumor-derived factors include activin and induce further expression and release of activin as well as a decrease of follistatin expression and its release by muscle cells, ultimately leading to myotube atrophy, a block of myogenic differentiation and hampered incorporation of myoblasts into myotubes. On the other hand, mechanical stimulation counteracts the negative effects exerted by tumor-derived factors on muscle cells by diminishing the levels of activin available to bind actRIIB: this is obtained by reducing activin concentration in the medium and by rescuing follistatin release by muscle cells. Recombinant activin (rActivin A) mimics tumor CM and its effects are counteracted by recombinant follistatin (rFollistatin). However, rFollistatin only partially counteracts CM: since, in the presence of CM, follistatin rescues the fusion index but not myotube diameter nor the number of NpM, while mechanical stimulation also reverts CM-mediated effects on myotube size, follistatin is mostly responsible for the regulation of myogenic differentiation, while mechanical stimulation preserves myotube size through additional mechanisms. The signaling pathways downstream of actRIIB involve the activation of SMAD2/3 transcriptional activity, which is increased by tumor-derived factors and decreased by mechanical stimulation, resulting in the regulation of MRF expression leading to myoblast differentiation and fusion.

Act receptor ligands are becoming increasingly important as triggers of muscle wasting and as pharmacological targets to treat cachexia. The myostatin-activin-SMAD cascade has been shown to activate FOXO3a, a crucial activator of muscle-atrophy-related gene expression (Mathew, 2011); ActRIIB antagonism suffices to revert muscle wasting and prolong survival in animals affected by cancer cachexia (Zhou et al., 2010). Additional studies highlighted activin relevance to humans, since increased circulating concentrations of activin may contribute to the development of cachexia in cancer patients (Loumaye et al., 2015). Here we showed that activin is present in the tumor-conditioned medium, inducing myotube atrophy and inhibiting the incorporation of myoblasts into nascent myotubes. We found that a mechanical stimulation-dependent rescue of the myotube size is indeed associated to an increase in the follistatin/activin ratio, showing that this is an effective *in vitro* model to identify beneficial muscle derived factors. In addition, CM seems to promote the release of activin from myotubes -inducing a vicious circle ultimately leading to myotube atrophy and hampered myotube growth - while mechanical stretching appears to diminish activin levels and increase the levels of the activin inhibitor follistatin. However, follistatin *per se* is not sufficient to fully revert CM negative effects, since recombinant follistatin only rescues FI without affecting myotube size (both in terms of diameter and recruitment of additional nuclei). As a consequence, additional factors in the tumor CM control myotube size and their negative effects are hampered by mechanical stimulation independently of follistatin release from myotubes. A model of the action of mechanical stimulation combined with tumor-derived factors on the release of activin and follistatin from myotubes is shown in Figure 3. In this context, actRIIB plays a major role, since its expression does not significantly change, through the activin-mediated SMAD2/3 transcriptional activity. The ratio between available activin and follistatin is likely the major player in these responses, even though the mechanisms underlying activin availability and the inhibition of activin by follistatin remain to be elucidated.

In conclusion: (a) the development of novel activin-targeted therapeutic approaches should consider the existence of further significant tumor-secreted factors mediating cachexia, even though activin plays a major role; (b) upon mechanical stimulation myotubes activate other pathways in addition to follistatin, which effectively counteract the adverse effect of tumor-derived factors; (c) in particular, in the presence of tumor-derived factors follistatin alone is not sufficient to recruit additional cells (nuclei) toward the myotubes, even though it increases the fusion index, representing the formation of new myotubes. *In vivo* muscle acts as a secretory organ (Pedersen, 2013); our results suggest that the pleiotropic effects of exercise are not limited to contraction-dependent endocrinological effects and that pure mechanical stimuli have a direct and relevant effect on muscle homeostasis.

## Author Contributions

AB, AB-S, and ZX performed the experiments and collaborated to the writing of the manuscript. MR performed the statistical analysis and contributed to the interpretation of the data and discussion. VM, MS, ZL, and SA supervised the experimental work, contributed to discussion, and revised the manuscript. DC supervised and coordinated the experimental work, contributed to data analysis, and wrote the first draft of the manuscript.

## Conflict of Interest Statement

The authors declare that the research was conducted in the absence of any commercial or financial relationships that could be construed as a potential conflict of interest.

## Supplementary Material

FIGURE S1 **(A)** Immunofluorescence for Myosin (red) in C2C12 myotubes at 6d of culture, following 2d of treatment with 10 ng/ml recombinant activin (rActivin), 25 ng/ml recombinant follistatin (rFollistantin) or both, with daily changes of medium. **(B)** Morphometric analysis was performed on replicate samples (*n* = 7) indicating a significant effect of both treatments and a negative interaction between them. Two-way ANOVA showed: significant effects on myotube diameter for activin (*F* = 7.72; *df* = 1; *p* < 0.05), follistatin (*F* = 4.35; *df* = 1; *p* < 0.05) and an interaction of follistatin with activin (*F* = 7.91; *df* = 1; *p* < 0.01); significant effects on fusion index for activin (*F* = 4.68; *df* = 1; *p* < 0.05), follistatin (*F* = 9.1; *df* = 1; *p* < 0.01). ^#^*p* < 0.05 ^##^*p* < 0.01 by Tukey HSD test. # *p* < 0.05 by Tukey HSD test.
