# Supplementary figures and images for: The Mechanical Stimulation of Myotubes Counteracts the Effects of Tumor-Derived Factors Through the Modulation of the Activin/Follistatin Ratio
Source: Front Physiol. 2019 Apr 24;10:401. doi: 10.3389/fphys.2019.00401 (PMC6491697; doi:10.3389/fphys.2019.00401)

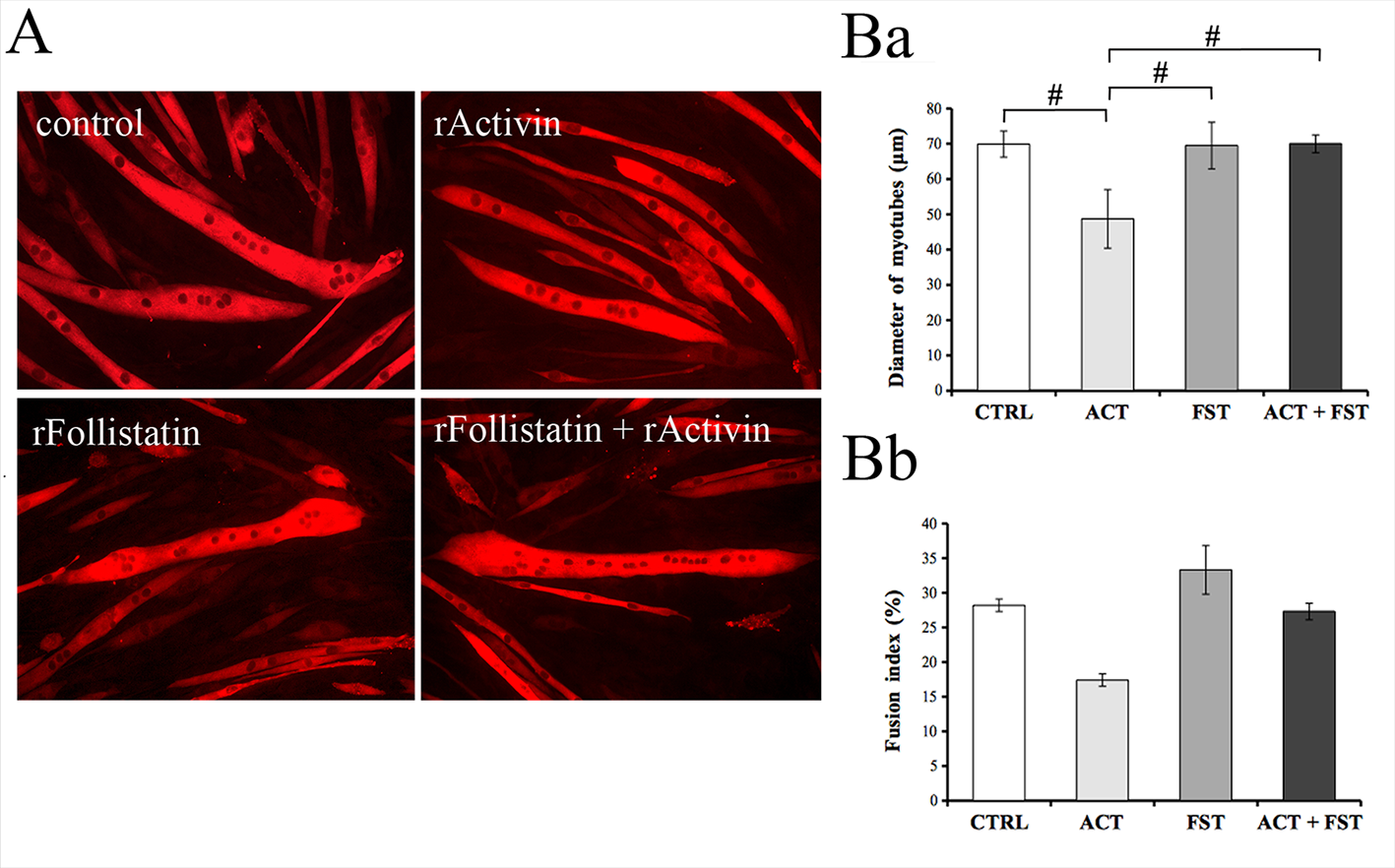

Supplement: FIGURE S1 — (A) Immunofluorescence for Myosin (red) in C2C12 myotubes at 6d of culture, following 2d of treatment with 10 ng/ml recombinant activin (rActivin), 25 ng/ml recombinant follistatin (rFollistantin) or both, with daily changes of medium. (B) Morphometric analysis was performed on replicate samples (n = 7) indicating a significant effect of both treatments and a negative interaction between them. Two-way ANOVA showed: significant effects on myotube diameter for activin (F = 7.72; df = 1; p < 0.05), follistatin (F = 4.35; df = 1; p < 0.05) and an interaction of follistatin with activin (F = 7.91; df = 1; p < 0.01); significant effects on fusion index for activin (F = 4.68; df = 1; p < 0.05), follistatin (F = 9.1; df = 1; p < 0.01). #p < 0.05 ##p < 0.01 by Tukey HSD test. # p < 0.05 by Tukey HSD test. [file Image_1.tiff]
